# Complete Genome Sequence of Staphylococcus edaphicus Strain CCM 8731

**DOI:** 10.1128/mra.00518-22

**Published:** 2022-08-10

**Authors:** Callum O. Rimmer, Jonathan C. Thomas

**Affiliations:** a School of Science and Technology, Nottingham Trent University, Nottingham, United Kingdom; University of Rochester School of Medicine and Dentistry

## Abstract

Here, we present the complete genome sequence of Staphylococcus edaphicus strain CCM 8731, which was originally isolated from Ross Island, Antarctica. The 2,749,487-bp sequence contains 2,709 predicted genes, with a G+C content of 33.4%. The complete genome was assembled using a hybrid approach with Oxford Nanopore Technologies long-read sequencing and Illumina short-read sequencing.

## ANNOUNCEMENT

Staphylococcal species have been isolated from a diverse array of human and environmental sources; however, few have been isolated from extreme environments (1). A complete genome for this species will accurately depict genome organization within the strain and serve as a high-quality reference for future studies (2).

Staphylococcus edaphicus strain CCM 8731 was isolated and cultivated by Pantůček et al. (1). We obtained S. edaphicus CCM 8731 as a freeze-dried sample from the Czech Collection of Microorganisms (http://www.sci.muni.cz/ccm). The strain was revived in Trypticase soy broth (Oxoid) and streaked onto Trypticase soy agar (TSA) for single colonies. A colony was transferred to 10 mL of brain heart infusion broth (Oxoid) and incubated overnight at 37°C. Bacterial cells were suspended in 200 μl of buffer EB (Qiagen) and lysed by incubation at 37°C with 80 μl of lysostaphin (240 units; Sigma). High-molecular-weight genomic DNA was extracted using Qiagen 100/G Genomic-tip columns.

Illumina library preparation was performed using the standard Illumina Nextera XT protocol, and the library was sequenced on a MiSeq system using v2 chemistry for 250-bp paired-end reads, producing approximately 110 Mbp of raw data (221,353 paired-end reads). Nanopore library preparation was performed using the ligation sequencing kit SQK-LSK109 (Oxford Nanopore Technologies) and native barcoding, with the library loaded onto a Flongle flow cell (R9.4.1). DNA was size selected using AMPure XP beads (Beckman Coulter, UK) but was not sheared. Approximately 145.5 Mbp of raw data was generated (21,236 reads, with an *N*_50_ value of 12.3 kbp).

Sequencing reads were assembled using default parameters for all software except where otherwise noted. Fast5 sequencing reads were basecalled using Guppy v3.6.1 (Oxford Nanopore Technologies). Adapter sequences were removed using Porechop v0.2.3 (3) with middle and end thresholds of 85% and 95%, respectively. Filtlong v0.2.1 (4) was used to filter reads based on quality and length. Overlapping reads were assembled into one contiguous sequence using Canu v1.4 (5). This assembly was polished using four iterations of Racon v1.4.2 (6) followed by Medaka v1.5 (7) and Nanopolish v0.13.2 (8). Illumina sequencing reads were trimmed using Trimmomatic v0.39 (9) to remove adapter sequences, leaving 160,478 trimmed paired-end reads. The Nanopolish-outputted genome was then error corrected with trimmed Illumina reads using Racon and Pilon v1.24 (10). The genome was manually trimmed and reorientated to *dnaA* with Circlator v1.5.5 (11) to produce a 2,749,487-bp assembly with a final combined coverage from Nanopore and Illumina data of 47.3×.

The complete genome was annotated using the NCBI Prokaryotic Genome Annotation Pipeline (PGAP) v6 (12), which predicted 2,709 genes, 19 rRNAs, and 61 tRNAs. Assembly quality was determined by CheckM v1.1.3 (13), with a G+C content of 33.4% and completeness of 99.45%. Average nucleotide identity (ANI) was calculated using fastANI v1.1 (14), showing 99.92% similarity to S. edaphicus CCM 8730^T^ and 85.44% similarity to its closest relative, Staphylococcus saprophyticus ATCC 15305^T^.

### Data availability.

All data are available under BioProject accession number PRJNA808309 and BioSample accession number SAMN26082960. The genome was deposited under GenBank accession number CP093217. Illumina raw read data and Nanopore base-called data were submitted to SRA and are available via accession numbers SRR18070734 and SRR18070733, respectively.
